# Considerations for Docking of Selective Angiotensin-Converting Enzyme Inhibitors

**DOI:** 10.3390/molecules25020295

**Published:** 2020-01-11

**Authors:** Julio Caballero

**Affiliations:** Centro de Bioinformática y Simulación Molecular (CBSM), Universidad de Talca, 1 Poniente No. 1141, Casilla 721, Talca 3460000, Chile; jcaballero@utalca.cl; Tel.: +56-712-418-850

**Keywords:** angiotensin-converting enzyme inhibitors, docking, interaction fingerprints, drug selectivity, rational drug design

## Abstract

The angiotensin-converting enzyme (ACE) is a two-domain dipeptidylcarboxypeptidase, which has a direct involvement in the control of blood pressure by performing the hydrolysis of angiotensin I to produce angiotensin II. At the same time, ACE hydrolyzes other substrates such as the vasodilator peptide bradykinin and the anti-inflammatory peptide N-acetyl-SDKP. In this sense, ACE inhibitors are bioactive substances with potential use as medicinal products for treatment or prevention of hypertension, heart failures, myocardial infarction, and other important diseases. This review examined the most recent literature reporting ACE inhibitors with the help of molecular modeling. The examples exposed here demonstrate that molecular modeling methods, including docking, molecular dynamics (MD) simulations, quantitative structure-activity relationship (QSAR), etc, are essential for a complete structural picture of the mode of action of ACE inhibitors, where molecular docking has a key role. Examples show that too many works identified ACE inhibitory activities of natural peptides and peptides obtained from hydrolysates. In addition, other works report non-peptide compounds extracted from natural sources and synthetic compounds. In all these cases, molecular docking was used to provide explanation of the chemical interactions between inhibitors and the ACE binding sites. For docking applications, most of the examples exposed here do not consider that: (i) ACE has two domains (nACE and cACE) with available X-ray structures, which are relevant for the design of selective inhibitors, and (ii) nACE and cACE binding sites have large dimensions, which leads to non-reliable solutions during docking calculations. In support of the solution of these problems, the structural information found in Protein Data Bank (PDB) was used to perform an interaction fingerprints (IFPs) analysis applied on both nACE and cACE domains. This analysis provides plots that identify the chemical interactions between ligands and both ACE binding sites, which can be used to guide docking experiments in the search of selective natural components or novel drugs. In addition, the use of hydrogen bond constraints in the S_2_ and S_2_′ subsites of nACE and cACE are suggested to guarantee that docking solutions are reliable.

## 1. Introduction

Angiotensin-I converting enzyme (EC 3.4.15.1; ACE) is a two-domain dipeptidyl carboxy-peptidase, which plays a key action in the renin-angiotensin system (RAS) and kallikrein-kinin system (KKS) involved in blood pressure regulation [1]. ACE hydrolyzes angiotensin I to yield the potent vasoconstrictor angiotensin II, and it also hydrolyzes the vasodilator bradykinin (BK) and the anti-inflammatory peptide N-acetyl-SDKP. Due to these biological actions, it is well known that ACE inhibition could lead to major clinical benefits in patients with hypertension, heart failures, myocardial infarction, and other related diseases. Structurally, ACE contains two homologous domains nACE and cACE that have a similar sequence and three-dimensional (3D) structure, but are characterized by a different pharmacological profiles, with distinctive specificities for substrates.

The development of ACE inhibitors was guided by a rational drug design protocol, defined as a logical process whereby molecules are constructed for precise fit with a macromolecular receptor’ [2]. Under this process, Cushman, Ondetti, and colleagues discovered in the seventies captopril, the first orally-active ACE inhibitor [2,3], which was approved by the United States Food and Drug Administration (FDA) in 1981 [4]. Captopril was discovered after structure-activity relationship (SAR) experiments of analogs of a peptide isolated from the venom of the Brazilian snake *Bothrops jararaca*, which was previously identified as the first competitive ACE inhibitor [5,6]. SAR experiments allow to identify that the C-terminal tripeptide WAP was optimal for interaction with ACE binding site. Modifications on WAP, with the help of a theoretical model of the binding site of carboxypeptidase A (enzyme similar to ACE), led to non-peptide inhibitors including the best candidate captopril [2,3].

Captopril is a mercaptoalkanoyl, a peptidomimetic with its thiol group forming a strong interaction with the central Zn^2+^ ion of ACE. Transformations of captopril led to enalapril, lisinopril [7], and a group of dicarboxylate derivatives patented and approved for medical use since more than twenty years ago (e.g., benazepril, perindopril, quinapril, ramipril, trandolapril, moexipril, etc) [8].

The number of ACE inhibitors approved for medical use is a very good example of the utility of rational drug design methods based on the knowledge of receptor structures with the help of molecular modeling protocols. However, ACE inhibitors designed until today are far from perfect; a number of adverse side effects have been described for them [9]. ACE inhibitors are used for controlling elevated blood pressure, preventing strokes, treating left ventricular dysfunction, congestive heart failure, nephropathy (in people with diabetes or hypertension), etc, but their common adverse effects include persistent cough, headache, dizziness, weakness, increased uric acid levels, etc.

It is well known that the lack of selectivity of ACE inhibitors is the cause of the adverse side effects due to progressive increments of BK in the patients [10]. Selective ACE inhibitors could be able to block the activities of one of the two homologous domains nACE or cACE. Despite both domains hydrolyze BK [11], their biological functions are different since angiotensin I hydrolysis depends exclusively on cACE [12] and the anti-inflammatory peptide N-acetyl-SDKP is hydrolyzed by nACE [13]. Therefore, it is predictable that selective cACE inhibitors decrease the production of angiotensin-II, while BK is normally hydrolyzed, with reduction of BK-mediated angioedema [14]. As a result, attenuation of vasodilator-related side effects could be obtained when the blood pressure is controlled by selective cACE inhibition [15]. On the other hand, selective inhibition of nACE increases the levels of N-acetyl-SDKP, leading to desired effects in prevention of cardiac, vascular, and renal inflammation and fibrosis [16]. The selective inhibition of nACE does not prevent the action of the other domain cACE (mainly the hydrolysis of BK), which contributes to normal regulation of blood pressure.

Regrettably, ACE inhibitors in use today to treat heart-related condition are non-selective. They are related to enalapril, which was designed too many years ago, when the differences in the biochemical profile of both ACE domains and the benefits of selective inhibition were still unknown. Molecular modeling methods were essential for the design of the first ACE inhibitors, and they could be essential in the design of the new generation of ACE inhibitors with selectivity. For this, the analysis in detail of nACE and cACE crystallographic structures is mandatory.

## 2. Rational Design of ACE Inhibitors and Crystallographic Structural Information

### 2.1. Structure of Both ACE Domains

**Figure 1:**
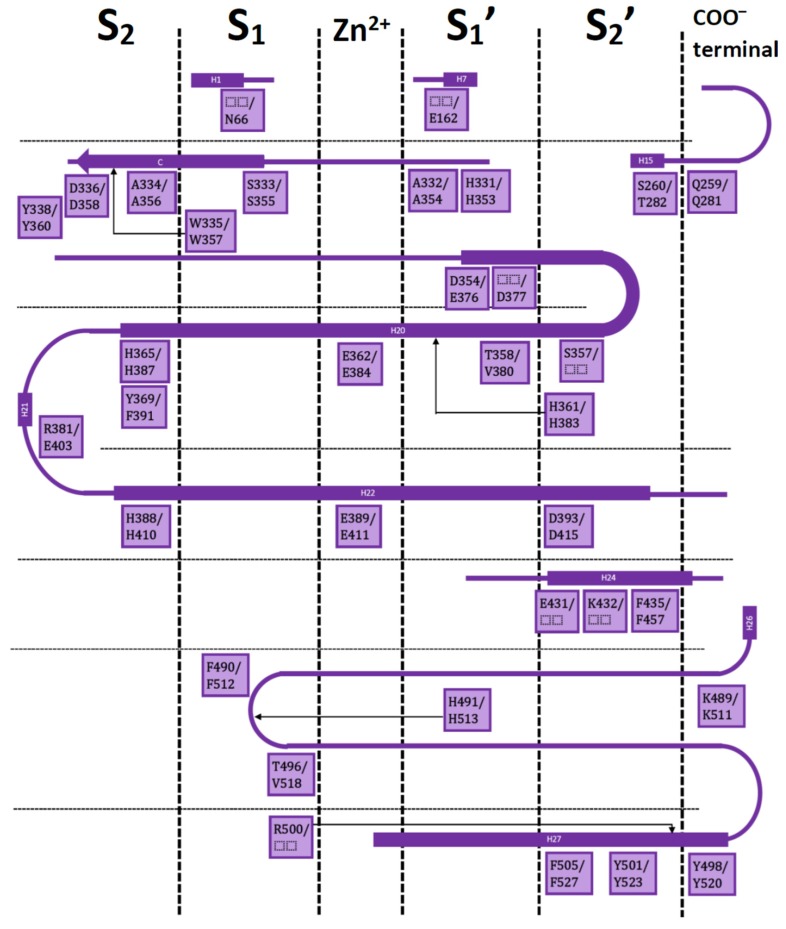
Residues in the S_2_, S_1_, S_1_′, and S_2_′ subsites with respect to the catalytic Zn^2+^ for nACE/cACE and their position in the sequences. Squares have nACE residues before the slash and cACE residues after the slash. Only residues from the IFP analysis are included.

Both ACE domains are very similar: with an ellipsoid-shaped structure, traversed by a long and deep active-site cleft, and with a preponderance of α-helices. The binding cavity is covered by a lid and contains a Zn^2+^ ion in the middle; four subsites denoted as S_2_, S_1_, S_1_′, and S_2_′ are on each side of the central ion (Figure 1).

Residues in these subsites are almost the same, but there are some subtle differences which are critical for substrate and inhibitor selectivity [17]. A comprehensive analysis on the available X-ray crystallographic structures of nACE and cACE allows to identify the residues involved in ligand binding, and the ones involved in differential binding (Figure 1). This information is indispensable for rational design of novel selective ACE inhibitors.

A good understanding of the chemical and shape requirements for selective ligands was reflected in the design of the phosphinic peptides RXP407 [18] and RXPA380 [19], which are the most potent nACE and cACE selective inhibitors to the present day. Both inhibitors have very specific interactions in the four subsites, but their selectivity is mainly due to specific interactions in the S_2_ subsite [20].

RXP407 is 2000-fold more selective for nACE. Its aspartate group is placed inside the S_2_ subsite of nACE forming a salt bridge with the residue Arg381 and hydrogen bond (HB) with Tyr369 (PDB code 3NXQ). These residues, Arg381 and Tyr369, are replaced in cACE by Glu403 and Phe391, respectively. On the other hand, RXPA380 is 2000-fold more selective for cACE. Its oxybenzyl group is placed inside the S_2_ subsite of cACE forming specific hydrophobic interactions with the residue Phe391 (PDB code 2OC2). These hydrophobic interactions lose their specificity in nACE due to replacement of Phe391 by Tyr369. The discovery of these drugs suggets that the most rational strategy for designing selective ACE inhibitors is the inclusion of groups to improve interactions in the S_2_ subsite. It is noteworthy that ACE inhibitors approved until today by FDA, such as enalapril and its related drugs, do not contain groups placed at this subsite. Therefore, a suitable strategy to develop new generation ACE inhibitors should incorporate: i) the structural features of previous successful inhibitors, which refers to the previously described contacts within ACE binding site, and ii) specific interactions in S_2_ subsite, which is the region with the more adequate pattern for providing selectivity to inhibitors. The use of molecular modeling methods is advantageous for accomplishing this strategy.

### 2.2. Molecular Modeling Applied to the Study of ACE Inhibitors in the Last Years

Molecular modeling methods are routinely used to investigate ACE inhibitors. In this review I want to illustrate these applications with very recent examples. The vast majority of reports on the discovery of ACE inhibitors that use any molecular modeling method are focused to the identification of small peptides extracted from natural sources or from protein hydrolysates and in silico methods, mainly molecular docking, are used to get insight into the structural features of the identified peptides [21,22,23,24,25,26,27,28,29,30,31,32,33,34,35,36,37,38,39,40,41,42,43,44,45,46,47,48,49,50,51,52,53,54,55,56].

For instance, Ashok et al. [21] identified bioactive peptide sequences from fat globule membrane protein (FGMP) hydrolysates of buffalo colostrum using a combination of empirical, computational and in vitro methods. They identified 89 FGMP peptides which were profiled for bioactivity and analyzed lead peptides by molecular docking for the inhibitory potential of ACE and dipeptidyl peptidase-4 (DPP-4). Finally, they found a heptapeptide (*m*/*z* 23.3) that inhibits ACE (IC_50_: 74.27 mM) and DPP-4 (IC_50_: 3.83 mM). In other work, Ugwu et al. [22] reported that camel and horse milk casein hydrolysates obtained from pepsin and trypsin combined enzymes exhibited ACE-inhibitory activity. They also performed docking of in silico generated fragments to propose the peptides from hydrolysates responsible for ACE inhibition. In other recent report, Xie et al. [23] identified that peptides TTW and VHW from digestion of *Chlorella vulgaris* proteins are ACE inhibitors. Authors used docking to study the mode of action of both peptides. In other work, Liu et al. [24] found a hydrophilic peptide (RYL) derived from agricultural waste (silkworm excrement and pupa) with high ACE-inhibitory activity. Authors used docking to study interactions between RYL and its target. In other work, Priyanto et al. [25] identified new ACE inhibitory peptides (VY-7 and VG-8) from a thermolysin digest of bitter melon (*Momordica charantia*) seed proteins. They used docking to study the orientation and chemical interactions of these peptides forming complexes with ACE. In other recent report, Yu et al. [30] identified three novel ACE inhibitory peptides EGF, HGR, and VDF derived from *Oncorhynchus mykiss* nebulin by in silico methods and tested their activities in vitro. Authors simulated the hydrolysis of nebulin and predicted online activity, solubility, absorption, distribution, metabolism, excretion, and toxicity (ADMET) properties of generated peptides. They performed docking experiments and observed that the peptides EGF, HGR, and VDF were docked into the S_1_ and S_2_ subsites of ACE. In other work, Yu et al. [31] identified nine novel ACE inhibitory peptides derived from *Todarodes pacificus* by using an in silico screening method. Firstly, authors found 126 peptides by simulated hydrolysis and they screened 30 peptides after predicting toxicity, allergenicity, gastrointestinal stability, and intestinal epithelial permeability. They observed that 21 peptides had been previously reported and nine were new. Authors synthesized these nine novel peptides to evaluate their in vitro ACE inhibition, showing IIY and NPPK had strong effects. Finally, they explored their interaction mechanisms and bonding configurations with ACE by using docking and molecular dynamics (MD) simulations. In other interesting report, Lin et al. [32] hydrolyzed Qula casein derived from yak milk casein and screened high ACE inhibitory activity peptides by using quantitative structure–activity relationship (QSAR) modeling integrated with molecular docking analysis. On the basis of the QSAR modeling predictions, authors selected a total of 16 peptides for molecular docking analysis and their docking study revealed that four of the peptides (KFPQY, MPFPKYP, MFPPQ, and QWQVL) bound the active site of ACE. Finally, authors synthesized these four novel peptides and they identified that KFPQY showed the highest ACE inhibitory activity. In other work, Li et al. [33] prepared efficient ACE-inhibitory peptides from sea cucumber-modified hydrolysates by adding exogenous proline. When proline was added, authors found that the modified hydrolysates exhibited higher ACE-inhibitory activity than the original hydrolysates. Among the modified hydrolysates, they identified two novel efficient ACE-inhibitory peptides PNVA and PNLG. Finally, they used docking to study the mode of action of their novel peptides.

Other recent works on the identification of natural peptides are mentioned here: Ali et al. [26] investigated the most likely binding poses of the decapeptide LVV-hemorphin-7 (which is the most stable form of hemorphin) with three biological targets, including ACE. They studied mammalian and camel LVV-hemorphin-7 and used computational methods (including docking and MM/GBSA) to calculate the binding affinities, and MD to gain a better understanding of the dynamics of the molecular interactions between the selected targets and hemorphin peptides. Their computational protocol helped to explain that camel hemorphin had a higher binding affinity for ACE when compared to the mammalian one. In other work, Wu et al. [27] identified three ACE inhibitory peptides, QLVP, QDVL, and QLDL, which account for the antihypertensive activity of *Ganoderma lucidum*, a polypore mushroom widely used in Asia to treat hypertension. They used docking and MD simulations to study the mode of action of the peptides. In other recent work, Gao et al. [28] isolated the ACE inhibitory dipeptides CC and CR from *Xerocomus badius* fermented shrimp processing waste. Authors used docking to explain the different activity between them (CC 100-fold more potent than CR). In other interesting work, Taga et al. [29] investigated the ACE inhibitory activities of X-Hyp-Gly-type tripeptides and they observed strong inhibition for Leu-Hyp-Gly, Ile-Hyp-Gly, and Val-Hyp-Gly. They also found that substitution of Hyp with Pro dramatically decreases inhibitory activity of X-Hyp-Gly, indicating that Hyp is important for ACE inhibition. They used molecular docking experiments using Leu-Hyp-Gly/Leu-Pro-Gly models to support these results. In other work, Setayesh-Mehr and Asoodeh [52] identified the peptides YLYELAR (HL-7) and AFPYYGHHLG (HL-10) from scorpion venom of *Hemiscorpius lepturus*. Authors found that these peptides are ACE inhibitors and performed docking experiments in nACE and cACE, suggesting a preference for the N domain. The same group (Kharazmi-Khorassani et al. [53]) studied the antioxidant property and ACE inhibition of thymosin alpha-1 (Thα1) peptide. Authors performed docking experiments in nACE and cACE, suggesting a preference for the N domain. In other work, Savitha et al. [54] reported the ACE-inhibitory potential of the tripeptide L-Phe-D-His-L-Leu in vitro and its antihypertensive effect in rat model of dexamethasone-induced hypertension. They performed docking experiments to study the mode of action of the studied peptide.

There are some theoretical reports on the use of molecular modeling to investigate peptide-ACE complexes. For instance, Jiang et al. [57] studied the different activities of the ACE-inhibitory peptides TFPHGP and HWTTQR extracted from hydrolysis of the seaweed pipefish by performing molecular docking and MD simulations. In other work, Qi et al. [58] used 3D-QSAR and docking to study the bioactivities of ACE-inhibitory peptides with phenylalanine C-terminus. According to the established models, they identified four novel ACE-inhibitory tripeptides GEF, VEF, VRF, and VKF, which displayed micromolar activities. The same authors (Qi et al. [59]) investigated the interactions between the bioactive peptide GEF and both ACE domains by using docking and MD simulations. In other work, Fang et al. [60] studied the interactions between three bioactive peptides (LIVT, YLVPH, and YLVR) and ACE by using docking and MD simulations. Authors results provide a theoretical basis for the design of ACE inhibitors as well as insight into the structural and molecular properties of ACE and the mechanisms of inhibition of the different peptide inhibitors [60]. In other work, Tong et al. [61] calculated amino acid descriptors and performed QSAR models applyed to 55 ACE tri-peptides inhibitors. Finally, Deng et al. [62] constructed a benchmark data set containing 141 unique ACE inhibitory dipeptides through database mining, and carried out a QSAR study to predict activities of potential novel peptides.

Other reports on the discovery of non-peptide natural products as ACE inhibitors also used molecular modeling methods [63,64,65,66,67]. For instance, Forero et al. [63] extracted and isolated two spermine derivatives (*N*^1^,*N*^4^,*N*^8^-tris(dihydrocaffeoyl)spermidine and *N*^1^,*N*^8^-bis(dihydrocaffeoyl)-spermidine) from lulo (*Solanum quitoense* Lam.) pulp. They demonstrated that these compounds exhibit a strong ACE inhibitory activity by using in vitro analyses and in silico molecular docking. In other work, Salehabadi et al. [64] performed a comprehensive surface plasmon resonance (SPR) study besides enzymatic assay for discovery of new ACE inhibitors via screening of medicinal plants. Authors found that *Onopordum acanthium* L. had the greatest ACE inhibition activity among the set of compounds and its active compound onopordia is an ACE inhibitor at micromolar level; they also used docking analysis to support the binding of onopordia to the ACE active site. In other work, Maneesh and Chakraborty [65] studied the ACE inhibitory activities of four undescribed O-heterocyclic analogues. They used docking to study the mode of action of the compounds.

There are also theoretical reports on the use of molecular modeling methods to investigate complexes between ACE and non-peptide natural products. For instance, Ahmad et al. [68] used molecular docking to study the potential ACE inhibitory activities and interaction conformations of nine polyphenolic compounds from *Peperomia pellucida* (L) Kunth. In other work, Arya et al. [69] studied the mode of action of chemical constituents of *Clerodendrum colebrookianum* against the anti-hypertensive drug targets Rho-associated coiled-coil protein kinase (ROCK), ACE, and phosphodiesterase 5 (PDE5) by using docking and MD simulations. They reported the mode of action of acteoside and osmanthuside β6 as ACE inhibitors. Finally, Moorthy et al. [70] performed virtual screening studies with docking and pharmacophore methods on a natural products data set to investigate its inhibitory effect on cardiovascular targets such as renin (REN) and ACE. They identified the compound Nat-59 that has remarkable interaction with both the studied targets (ACE and REN). They also designed a lead compound NLC-1 on the basis of the pharmacophoric features provided by pharmacophore and docking studies. Finally, they performed MD simulations on the compound NLC-1 in order to find out the binding features of this molecule on the targets (ACE and REN).

Other examples show studies on the discovery of novel synthetic ACE inhibitors with the help of molecular modeling methods [71,72,73,74]. For instance, Saadaoui et al. [72] synthesized Schiff bases of 4-amino-1,2,4-triazole derivatives and evaluated them as ACE inhibitors. They observed that the synthetic compounds displayed virtually no cytotoxicity and they proposed the mode of action of the compounds by using in silico molecular docking. The same authors (Salah et al. [73]) synthesized a series of novel 1,2,4-triazolones and bistriazol-4-yl-2-yl pyridine derivatives and they found that they have shown significant inhibitory action against ACE. They proposed the orientations and chemical interactions of the compounds forming complexes with ACE by using molecular docking. In other work, Abouelkheir and El-Metwally [71] used docking-based screening to investigate the ability of different DPP-4 inhibitors or ACE inhibitors to interact with DPP-4 and ACE. They extrapolated the results of screening into animal study and they found that some DPP-4 inhibitors could inhibit ACE, which partially explains the cardio-renal effects of these drugs. In other work, Manikandan et al. [74] synthesized a family of 12 members of naphthalene-2-ol-indolin-2-one-thiocarbamides and they found that these compounds show inhibitory activity on ACE. They also investigated the binding modes of the designed compounds into both nACE and cACE active sites.

As an example of theoretical reports on the use of molecular modeling methods to investigate complexes between ACE and synthetic compounds, the work of Stoičkov et al. [75] can be mentioned. These authors developed QSAR models for a series of non-peptide compounds (63 derivatives of N-substituted (mercaptoalkanoyl)- and [(acylthio)alkanoyl] glycines) as ACE inhibitors by using descriptors based on Simplified Molecular Input-Line Entry System (SMILES) notation and local graph invariants. They also used molecular docking for the final assessment of the best QSAR model and the design of novel inhibitors.

The above examples show cases where molecular modeling methods were used for discovery of novel ACE inhibitors; however, they did not consider the discovery or design of nACE or cACE selective inhibitors. An interesting study was recently reported about the selectivity of sampatrilat and its derivative samAsp. Sampatrilat, a dual inhibitor of ACE and neutral endopeptidase [76], was found that is 12.4-fold more selective for cACE [77]. It occupies the S_2_ subsite of both ACE domains, which was demonstrated by molecular modeling methods [77] and later by crystallographic structure [78]. The moderate selectivity of sampatrilat for cACE was explained by electrostatic interactions between the lysine of the ligand and the residue Glu403 of cACE (replaced by Arg381 in nACE). The sampatrilat derivative samAsp was designed for searching for a better interaction with Arg381 of nACE and, therefore, selectivity for nACE. For this, samAsp contains an aspartate group instead the lysine of sampatrilat; however, samAsp is not selective and has lower micromolar affinity for both ACE domains [77].

Docking and MD simulations were used in the molecular modeling protocol which confirmed that the lysine group of sampatrilat could have favorable electrostatic interactions in the S_2_ subsite of cACE, which are lost in the S_2_ subsite of nACE [77]. The same protocol applied to samAsp forming complexes with nACE and cACE suggested that aspartate in samAsp is too short to form HB or electrostatic interactions with the residue Arg381 in nACE; instead, it is more close to other residues in the S_2_ subsite with no contribution to selectivity. This result gives a possible explanation to the experimental samAsp non-specific inhibition for ACE domains.

Although several differences were found, both molecular modeling and crystallographic determinations agreed in the global orientation of sampatrilat inside nACE and cACE [77,78]. Molecular modeling found the orientation S2 for sampatrilat, where the lysine side chain of the ligand is placed in the S_2_ subsite of both domains. In this position, the lysine group of sampatrilat is close to the residue Glu403 in S_2_ of cACE, forming electrostatic and hydrogen bond interactions; these interactions are lost when Glu403 is replaced by Arg381 in nACE [77]. Orientations S2 of sampatrilat in both domains were confirmed by crystallographic determinations (PDB codes 6F9V and 6F9R) [78]. Another orientations, named S1, were found when the lysine group of sampatrilat was placed near the residues Asn70 and Glu143 in S_1_ of cACE or residues Asp44 and Ser119 in S_1_ of nACE [77]. Orientations S1 were discarded by crystallographic determinations [78]. A key difference between orientations S2 and S1 is observed in the formation of HBs between sampatrilat and the backbone NH and CO of the cACE residue Ala356 (Ala334 in nACE) in orientation S2, which is not formed in orientation S1. Analysis of PDB structures suggests that interactions with this alanine are essential for selectivity of ACE inhibitors.

### 2.3. Molecular Modeling for the Development of Novel Selective Inhibitors

The group of Sturrock and collaborators has been focused to the design of novel selective ACE inhibitors since the last decade, with the help of molecular modeling methods in some instances. Five years ago, this group modified RXP407 backbone with different chemical substituents by using the receptor-based SHOP (scaffold hopping) de novo design method [79,80], yielding the highly selective tetrazole derivative 33RE [81]. Theoretically, 33RE has improved interaction energies with nACE residues which are different from that of cACE. Experimentally, it is a potent nACE inhibitor (Ki = 11.21 nM), 927-fold more selective for nACE than cACE. In other recent report [82], the same authors explored the Gunupati Venkata Krishna (GVK) Biosciences database [83] for finding ACE inhibitors with possible nACE selective binding patterns. They used docking and MM/GBSA methods to model diprolyl chemical series selected from the above mentioned database, and thereafter, they modified these compounds by including substituents to have interactions with nACE residues at S_2_ subsite. Theoretically and experimentally, they found an aspartate-modified diprolyl compound with favorable electrostatic interactions with the nACE residue Arg381 and with a potent nACE inhibition (Ki = 11.45 nM) and 84-fold more selectivity for nACE than cACE. These two examples include the use of structural information contained in PDB and simple molecular modeling protocols to guide in the design of novel selective ACE inhibitors.

## 3. Interaction Fingerprints (IFPs) for ACE Inhibitors Derived from PDB Structures

In this section I am focused on identifying the recurrent interactions between ACE domain binding sites and their ligands, and the residues involved in these interactions. Interaction fingerprints (IFPs) is a very effective method for such analysis, with recent successful applications in diverse protein-ligand systems [84,85,86,87]. “Interaction Fingerprints Panel” of Maestro (Maestro 10.2.011, Schrödinger LLC) was used for constructing the IFPs as described in Singh et al. [88,89]. The results of IFPs are the plots of % of occurrence of chemical interactions that represent a summary of the structural information of nACE/cACE-ligand complexes until today. Such information should be consider in rational design of novel selective ACE inhibitors.

IFPs capture chemical contacts between ligand groups and the residues in the protein binding site by establishing the following chemotypes: polar (P), hydrophobic (H), HBs where the residue is acceptor (A), HBs where the residue is donor (D), aromatic (Ar), and electrostatic interactions with charged groups (Ch). They also differentiate between contacts with backbone and contacts with side-chain functional groups.

The residues and their contributions as part of the identified chemotypes are described by considering the well-known scheme of the ACE binding site occupied by a conventional inhibitor. Conventional ACE inhibitors bind to the catalytic region of the active sites of nACE and cACE via a chelation with the central Zn^2+^, while the groups P_2_′, P_1_′, P_1_, and P_2_, mimicking substrate peptides, are placed inside the subsites S_2_′, S_1_′, S_1_, and S_2_, respectively.

First, IFPs were calculated by considering 11 nACE-inhibitor complexes reported in PDB (PDB IDs 2C6N, 2XYD, 3NXQ, 4BXK, 4BZS, 4CA6, 6EN5, 6EN6, 6F9R, 6F9V, and 6H5X), and then, IFPs were calculated by considering 13 cACE-inhibitor complexes reported in PDB (PDB IDs 1O86, 1UZE, 1UZF, 2OC2, 2XY9, 3BKK, 3BKL, 3L3N, 4BZR, 4CA5, 6F9T, 6F9U, and 6H5W).

**Figure 2:**
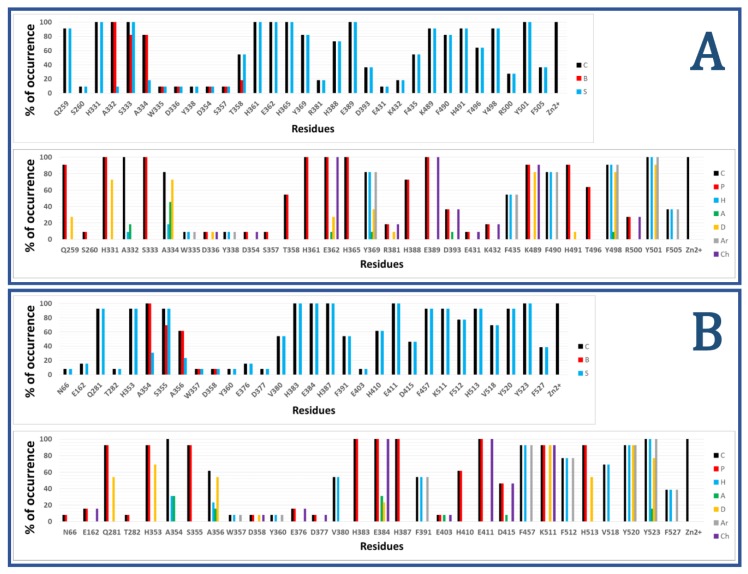
Occurrence of interaction types at the nACE and cACE binding interfaces from IFPs for ACE-ligand structures reported in PDB, (nACE-ligand structures are in (**A**) and cACE-ligand structures are in (**B**)). Top part of both A and B plots % of occurrence of contacts C, interactions with the backbone of the residue B, and interactions with the side chain of the residue S per residue. Bottom part of both A and B plots % of occurrence of chemical interactions: contacts C, polar P, hydrophobic H, HBs where the residue is acceptor A, HBs where the residue is donor D, aromatic Ar, and electrostatic with charged groups Ch.

Figure 2 shows the results of calculated IFPs for ACE inhibitors. The IFP analysis applied to the 11 nACE-inhibitor complexes reported in PDB revealed that 31 nACE residues had contacts with inhibitors (Figure 2a, also represented before the slash in squares in Figure 1). On the other hand, the IFP analysis applied to the 13 cACE-inhibitor complexes reported in PDB revealed that 30 cACE residues had contacts with inhibitors (Figure 2b, also represented after the slash in squares in Figure 1).

The plots of % of occurrence obtained from IFP calculations in Figure 2 show the importance of the residues that anchor the terminal carboxylate group of the ligands to ACE binding sites. The triad formed between the residues Q259/Q281 at the loop before the helix H15, K489/K511 at the loop after the helix H26, and Y498/Y520 at the beginning of the helix H27 of nACE/cACE has been identified as essential for binding affinity of ligands. The three residues have polar contributions in more than 90% of the total ACE-ligand structures in PDB. They also act as HB donors (Q259/Q281 in more than 25%/50%, K489/K511 in more than 80%/90%, and Y498/Y520 in more than 80%/90%, of the nACE/cACE-ligand structures, respectively). K489/K511 have also electrostatic contributions in more than 90% of the total ACE-ligand structures and Y498/Y520 also provide hydrophobic and aromatic contributions in more than 90% of the total ACE-ligand structures.

The plots of % of occurrence in Figure 2 also show the residues with the major contributions in S_2_′ of ACE binding sites. The residues F435/F457 at the helix H24, Y501/Y523 at the helix H27, and F505/F527 (also at H27) provide hydrophobic and aromatic contributions. Y501/Y523, at the entrance of the S_2_′ subsite, have these contributions in 100% of the total ACE-ligand structures; meanwhile, F505/F527, at the deeper part of the pocket, have these contributions in 30% of them. Lastly, F435/F457, also at the deeper part of the pocket, have these contributions in more than 50%/90% of the nACE/cACE-ligand structures, respectively. The residues Y501/Y523 also act as HB donors in more than 90%/75% of the nACE/cACE-ligand structures reported in PDB, respectively, and Y523 (cACE) acts as HB acceptor in more than 15 % of the cACE-ligand structures. The residues D393/D415 (helix H22), also at S_2_′ subsite, have polar and electrostatic contributions in more than 35%/45% of the nACE/cACE-ligand structures, respectively. They also can act as HB acceptors (e.g., in the complex between cACE and lisinopril-tryptophan (lisW) [90]). Finally, the residues H361/H383 (at the helix H20), which is coordinated to Zn^2+^, have also polar contributions to the S_2_′ subsite in 100% of the total ACE-ligand structures.

The residues with major contributions in S_1_′ of ACE binding sites are also found in the plots of % of occurrence obtained from IFP calculations (Figure 2). The residues H331/H353 at the loop before the β-sheet C and H491/H513 at the loop after the helix H26 have polar contributions. H331/H353 and H491/H513 have these contributions in more than 90% of the total ACE-ligand structures, and they act as HB donors (H331/H353: in around 70% of the total structures, and H491/H513: in more than 9%/50% of the nACE/cACE-ligand structures, respectively). It is important to highlight that these histidine residues (H331/H353 and H491/H513) anchor the CONH carbonyl group at P_1_′ position of substrate peptides, and they have the same role in the binding of inhibitors (as observed in PDB structures and identified by IFPs).

The residues A332/A354 (at the loop before the β-sheet C), also at S_1_′ subsite, provide hydrophobic contributions in 9%/30% of the nACE/cACE-ligand structures, respectively. They also act as HB acceptors in 18%/30% of the nACE/cACE-ligand structures, respectively.

The threonine residue T358 (at helix H20) in the S_1_′ subsite of nACE is changed by the valine residue V380 in the same subsite of cACE. T358 has polar contributions in 54.5% of the nACE-ligand structures. On the other hand, V380 has hydrophobic contributions in 53.8% of the cACE-ligand structures. This subtle difference, contributing to differential polarity between S_1_′ subsites of nACE and cACE, could be considered in the design of selective ACE inhibitors. According to Nchinda et al., the replacement of T358 (nACE) by V380 (cACE) explains the increase of cACE selectivity when the P_2_′ proline of lisinopril is changed by tryptophan in lisW [91]. The crystallographic structures of lisinopril-ACE complexes show that T358/V380 are in the S_1_′ subsite, close to the P_1_′ group lysine [92]; moreover, they are far from P_2_′ tryptophan in crystallographic structures of compounds RXPA380 [20] and kaW [93]. However, the P_2_′ tryptophan is oriented in a conformation different from that observed in RXPA380 and kaW in the crystallographic structure of the lisW-cACE complex [90]. In this structure, the P_2_′ tryptophan is close to V380, and this valine residue is intercalated between the P_1_′ lysine and P_2_′ tryptophan moieties; therefore, the replacement of V380 by T358 can affect the selectivity of lisW for both ACE domains.

The plots of % of occurrence in Figure 2 also show the contributions of residues close to the Zn^2+^ of ACE binding sites. The residues E362/E384 at the helix H20 and E389/E411 at the helix H22 have polar and electrostatic contributions in 100% of the total structures. E362/E384 also act as HB donors in more than 25%/20% of the nACE/cACE-ligand structures, respectively, and they act as HB acceptors in more than 9%/30% of the nACE/cACE-ligand structures respectively.

The residues with major contributions in S_1_ of ACE binding sites are also found in the plots of % of occurrence obtained from IFP calculations (Figure 2). The residues S333/S355 at the β-sheet C of nACE/cACE have polar contributions in more than 90% of the total structures. On the other hand, the residues F490/F512 (at the loop after the helix H26) provide hydrophobic and aromatic contributions in 80%/75% of the nACE/cACE-ligand structures, respectively.

The threonine residue T496 (at the loop after the helix H26) in the S_1_ subsite of nACE is changed by the valine residue V518 in the same subsite of cACE. T496 has polar contributions in 63.6% of the nACE-ligand structures. On the other hand, V518 has hydrophobic contributions in 69.2% of the cACE-ligand structures. This subtle difference, contributing to differential polarity between S_1_ subsites of nACE and cACE, has not been considered in the design of selective ACE inhibitors.

The plots of % of occurrence in Figure 2 also show the residues with contributions in S_2_ of ACE binding sites. The residues A334/A356 (at the β-sheet C) have contacts in more than 80%/60% of the nACE/cACE-ligand structures respectively. They have hydrophobic contributions in more than 15%/20% of the nACE/cACE-ligand structures respectively. They also act as HB donors in more than 70%/50% of the nACE/cACE-ligand structures respectively, and as HB acceptors in more than 45%/15% of the nACE/cACE-ligand structures respectively. It is important to highlight that the backbone NH and CO groups of these residues anchor the amino acid at P_2_ position of substrate peptides, and they have the same role in the binding of inhibitors (as observed in PDB structures and identified by IFPs).

The residues H365/H387 at the helix H20 and H388/H410 at the helix H22 have polar contributions. H365/H387, which is coordinated to Zn^2+^, have these contributions in 100% of the total structures, and H388/H410 have these contributions in 70%/60% of the nACE/cACE-ligand structures, respectively. The residues Y369/F391 at the end of the helix H20 of nACE/cACE provide hydrophobic and aromatic contributions in more than 80%/50%, of the nACE/cACE-ligand structures, respectively. Y369 also acts as HB donor in 36.4% and acts as HB acceptor in 9% of the nACE-ligand structures.

The residues R381/E403 have different effects due to their different charges. R381 has polar and electrostatic contribution of 18.2% and acts as HB donor in 9.1% of the nACE-ligand structures. On the other hand, E403 has polar and electrostatic contribution of 7.7% and acts as HB acceptor in 7.7% of the cACE-ligand structures. Commonly, the analysis of IFPs detect residues that are essential for the binding of ligands by means of identification of higher percent of occurrences; however, it is important to consider that most of the crystallized ligands (used to construct these IFPs) do not contain groups P_2_ at the S_2_ subsite. Therefore, the low percent of occurrence of S_2_ residues should be observed with caution. In fact, the residues R381/E403, with low percent of occurrences, have been essential for the design of selective ACE inhibitors as mentioned above.

## 4. Docking of Selective ACE Inhibitors is Facilitated by the Use of Constraints

Docking of ACE inhibitors is not an easy task due to the shape and large dimension of the binding sites. Docking experiments typically yield very different solutions that do not share the orientations of inhibitors inside ACE domains. These orientations could lead to wrong conclusions. As an example, the above mentioned work studying the binding of sampatrilat suggested that this drug could have two orientations: S1 and S2 [77], but crystallographic structures discarded S1 [78]. The orientation S1 seemed reasonable when a superficial analysis of ACE binding site is performed; however, it does not comply with a very important requirement detected by IFPs: HB interactions with the residues A334/A356 are important according to IFPs, and it is possible to see that they are in all the structures with groups inside the S_2_ subsite.

A strategy for increasing successful docking solutions is to include constraints that consider key HB interactions detected by IFPs. This is not a novel idea, constraints in docking of ACE inhibitors were previously used by Fienberg and collaborators in the modeling of diprolyl chemical series [82]. These authors executed a protocol including docking (with Glide method [94,95]) and MM/GBSA, and they indicated that docking programs do not efficiently recreate the native pose of ACE inhibitors in the binding site without the use of docking constraints. Therefore, they used three different constraint conditions to help find plausible docking poses. These conditions included positional (1Å spheres), metal chelation, and HB docking constraints. They defined three conditions C1, C2, and C3 including chelation between the zinc binding group of the ligand and the Zn^2+^ ion, and an additional constraint. C1 added HB with K489/K511 or Q259/Q281, C2 added amide/amine N in a 1Å constraint sphere around Cα of group P_2_′, and C3 had the same additional constraint of C2 or alkyl C in a 1Å constraint sphere located close to Cα of group P_1_ (more details in reference [82]). This strategy led to successful docking poses, with a plausible pose into the two nACE and cACE active sites using one of the three constraint conditions for the diprolyl compounds under study.

**Figure 3:**
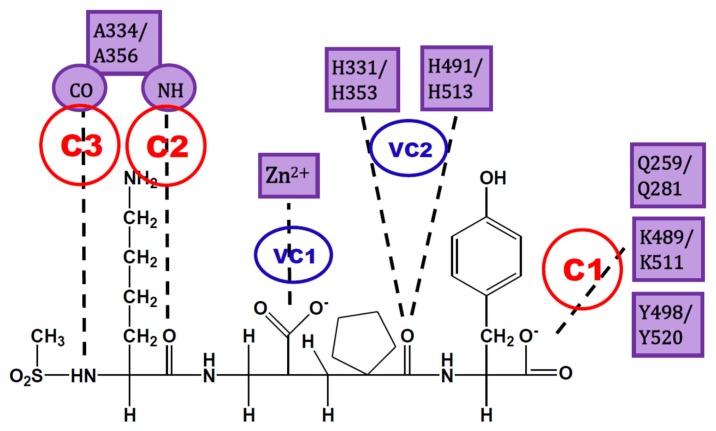
Constraints proposed for ACE inhibitors. C1, C2 and C3 are the constraints defined for docking experiment. VC1 and VC2 are constraints subsequently detected by visual inspection.

The analysis of IFPs could lead to a simple scheme of constraints to help find plausible docking poses. A simple option is the selection of essential HBs for ACE-ligand complexes according to the crystallographic information of nACE and cACE-inhibitor complexes in PDB (and processed by using IFPs). These HBs are described below and are represented in Figure 3:

C1: A C-terminal group of the ligand must be an HB acceptor to K489/K511 from nACE/cACE and it must have proximity to Q259/Q281 and Y498/Y520 from nACE/cACE.
VC1: The ligand must have an HB acceptor group at P_1_′ to form HB with H331 and/or H491 (nACE) or H353 and/or H513 (cACE). Both histidine residues in nACE and cACE must have a proton at position ε (this consideration should be important when molecular mechanics methods are used).
VC2: The ligand must have a polar group (a negatively charged group or an electron donor group) forming interactions with the Zn^2+^ ion.
C2: Ligand must be an HB acceptor to NH of Ala334 (nACE) or Ala356 (cACE)
C3: Ligand must be an HB donor to CO of Ala334 (nACE) or Ala356 (cACE).

All these constraints are needed, but in practice it is possible to “grab the rope by the ends”; therefore, I suggest to define constraints C1, C2, and C3, where C1 is mandatory and at least one of C2 and C3 is also essential. The visual constraints VC1 and VC2 are also mandatory, but they can be used after docking solution to corroborate the quality of the obtained pose. In short, constraints C1 and C2 (or C3) can be used to guide docking experiment, but the obtained pose must comply with VC1 and VC2 constraints subsequently detected by visual inspection (or an automated script). This procedure could provide enough feasibility during virtual screening processes, as a filter of plausible solutions without an excess of rigidity (a definition of too many constraints can lead to a very short number of solutions).

The HB constraint method was tested in the docking of the diprolyl derivatives **1**, **2**, and **3** from reference [82]. These compounds were docked in nACE (PDB codes 6EN5 and 6F9V) and cACE (PDB codes 6F9T and 6F9U) binding sites. Docking was performed with Glide SP and XP methods [94,95], by using the same protocol and parameters that were used by my group in previous works [96,97,98,99], and by including constraints C1 and C2 (or C3).

The results are reported in Table 1. It is possible to see that plausible poses were found for compounds **1**, **2**, and **3** guided by constraints C1 and C2 (or C3) and constraints VC1 and VC2 are also fulfilled. When the same docking experiment was done without constraint, a big number of non-plausible conformations were found. This test confirms that definition of constraints in an ACE-ligand docking protocol is needed, as reported previously, and I suggest that HB constraint strategy lead to plausible docking solutions as the one used by Fienberg et al. [82].

The use of constraints could contribute to the finding of selective inhibitors. They guarantee that all the essential HBs (defined by C1, C2, C3, VC1, and VC2) are found during docking process. If these essential HBs are in the right position, mainly the HBs defined by C1 and/or C2, the group P_2_ could have the right orientation to interact with the residues Y369/F391 and R381/E403 at the S_2_ subsite of nACE/cACE. These residues have been reported as essential for the high selectivity of compounds RXP407 [18], RXPA380 [19] and 33RE [81]. The PDB structures of these compounds show all the HBs defined by C1, C2, C3, VC1, and VC2; therefore, they could be considered as the essential chemical interactions described for analog ligands (ECIDALs) [100,101] for nACE/cACE-ligand complexes; it is expected that novel ACE inhibitors should comply with these ECIDALs.

In silico molecular docking methods could contribute to the design of a next generation of selective ACE inhibitors. However, good results of calculations will be guaranteed if the X-ray crystallographic information in PDB is considered. The recommendation in this section is to ensure that poses obtained from molecular docking should be similar to those in crystallized ACE-inhibitor complexes. The use of constraints during docking calculations is an easy way to reduce the number of non-adequate solutions, yielding rational poses, and improving the reliability of the in silico protocol for the design of more specific ACE inhibitors.

## 5. Conclusions

There are many examples, among the recent studies on the identification and design of ACE inhibitors that have used molecular docking for proposing the interactions between the inhibitors and the ACE binding site. Most of these reports identified natural peptides or peptides from hydrolysates that act as ACE inhibitors and lesser reports identified non-peptide natural products or synthetic compounds as ACE inhibitors. A comprehensive review of these studies showed that large dimensions of ACE binding site are not commonly considered for docking application. Besides, the information of previous structures available in PDB and the presence of the two domains nACE and cACE were also ignored in most studies.

In this work I defend the proposal that molecular docking calculations with constraints in both nACE and cACE binding sites are needed to investigate the mode of action of ACE inhibitors. With this in mind, IFP analysis was performed to nACE/cACE-inhibitor complexes structures in PDB and obtained percent of occurrences of the interactions between ligands and both nACE and cACE binding sites. IFPs describe the essential chemical interactions in the complexes and identify the key role of essential HBs. I propose that a good strategy is to define two HB constraints for docking calculation: (i) between the ligand and the residues K489/K511 from nACE/cACE and (ii) between the ligand and the residues A334/A356 from nACE/cACE. The docking solutions that contain HBs with H331 and/or H491 (nACE) or H353 and/or H513 (cACE) and a polar group forming metal–chelator interaction can be defined as reliable.

This report gives advice to researchers interested in reporting the mode of action of ACE inhibitors that they should consider the structural information of ACE-inhibitor complexes in PDB when docking calculations are executed. This information should be used to distinguish good solutions from the bad ones, and the final docking solution could be consider as reliable. Compliance with these considerations will increase the value of docking experiments for the characterization of ACE-ligand inhibitors and design of novel candidates. The use of constraints in docking of ACE-inhibitors is a good way to increase the number of good solutions.

## Figures and Tables

**Table 1:**
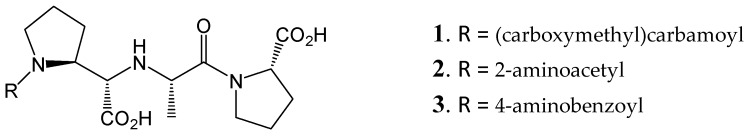
ACE-ligand HB distances for poses obtained from Glide docking using constraints C1 and C2 (or C3).

Compound	PDB	d[C3] (Å) ^1^	d[C2] (Å) ^2^	d[VC2] (Å) ^3^	d[VC1] (Å) ^4^	d[C1] (Å) ^5^
nACE						
**1**	(6EN5//6F9V)	-	3.1//3.2	1.9//2.0	2.9/2.9//2.7/2.9	2.7//3.0
**2**	(6F9V)	2.9	-	2.9	3.2/2.8	3.1
**3**	(6EN5)	-	3.1	1.9	2.9/2.8	2.9
cACE						
**1**	(6F9T//6F9U)	-	3.0//3.2	1.9//2.1	7.6 ^6^/3.2//4.1/2.7	2.8//2.8
**2**	(6F9U)	2.6	-	2.0	2.5/2.7	3.2
**3**	(6F9T)	3.1	-	1.9	8.6 ^6^/3.2	2.8

^1^ d[C3] represents the distance between one of the oxygen or nitrogen atoms from the ligand R group (the closest one) and CO of the residue A334/A356 from nACE/cACE. ^2^ d[C2] represents the distance between one of the oxygen atoms from the ligand R group (the closest one) and NH of the residue A334/A356 from nACE/cACE. ^3^ d[VC2] represents the distance between one of the ligand central carboxylate oxygen atoms (the closest one) and the Zn^2+^ ion. ^4^ d[VC1] represents the distance between the ligand CO oxygen atom and the Nε of the residue H331/H353 from nACE/cACE//and the distance between the ligand CO oxygen atom and the Nε of the residue H491/H513 from nACE/cACE. ^5^ d[C1] represents the distance between one of the ligand C-terminal carboxylate oxygen atoms (the closest one) and the side chain N of the residue K489/K511 from nACE/cACE. ^6^ High values of d[VC1] in PDB 6F9T are expected for distance between the ligand CO oxygen atom and the Nε of the residue H353 because an atypical displacement of the residue H353 in this structure.
